# Mini Review on the Structure and Properties (Photocatalysis), and Preparation Techniques of Graphitic Carbon Nitride Nano-Based Particle, and Its Applications

**DOI:** 10.1186/s11671-018-2702-3

**Published:** 2018-11-29

**Authors:** Williams Kweku Darkwah, Yanhui Ao

**Affiliations:** Key Laboratory of Integrated Regulation and Resource Development on Shallow Lakes, Ministry of Education, Environmental Engineering Department, College of Environment, Hohai University, Nanjing, China

**Keywords:** Photocatalysis, Graphite carbon nitride (g-C_3_N_4_), Carbon nitride nano-based particle

## Abstract

Graphite carbon nitride (g-C_3_N_4_) is well known as one of the most promising materials for photocatalytic activities, such as CO_2_ reduction and water splitting, and environmental remediation through the removal of organic pollutants. On the other hand, carbon nitride also pose outstanding properties and extensive application forecasts in the aspect of field emission properties. In this mini review, the novel structure, synthesis and preparation techniques of full-bodied g-C_3_N_4_-based composite and films were revealed. This mini review discussed contemporary advancement in the structure, synthesis, and diverse methods used for preparing g-C_3_N_4_ nanostructured materials. The present study gives an account of full knowledge of the use of the exceptional structural and properties, and the preparation techniques of graphite carbon nitride (g-C_3_N_4_) and its applications.

## Introduction

The central energy source elated from the extraterrestrial space, solar energy capacities to surpass the almanac world’s energy request by a large border [1]. Given the long forecast era of the Sun, solar energy is also considered the ultimate renewable source that can be harvested on the planet, Earth [2, 3]. The unending and discontinuous nature of this energy source, however, presents key challenges in relationships of harvesting, storage, and utilization [4]. At the moment, there are a measure of technologies in place that may be used to face them. Solar energy can be flexibly gathered, transformed and kept in the form of heat, which can either distribute heat to residence or be further converted into electricity, as well as into other forms of energy [5]. The most innovative investigated technologies concerning solar photon gaining may be on those by the photocatalysis, as described by Edmond Becquerel, 1839 [5].

Predominantly, wastewater is the major source of pollution, specifically, wastewater produced due to chemical industrialization, because this wastewater contains pronounced concentration of large organic fragments which are tremendously poisonous and carcinogenic in nature [3]. Previously, the environmental remediation technology (which comprises of adsorption, biological oxidation, chemical oxidation, and incineration) has been used in the treatment of all types of organic and toxic wastewater and also has its effective application in solar energy utilization, environmental treatment, and biomedical and sensing applications. Fujishima and Honda revealed the exceptional knowledge about the photochemical splitting of water into hydrogen and oxygen in the presence of TiO_2_ in 1972; research interest has been focused in heterogeneous photocatalysis [3–5]. The speeding up of photoreaction in the existence of a catalyst is described as photocatalysis. Photocatalysis reaction is best known to be carried out in media such as gas phase, pure organic liquid phases, or aqueous solutions. Also, in most chemical degradation methods, photocatalytic degradation vis-à-vis photons and a catalyst is often identified as the best in controlling of organic wastewater, solar energy utilization, environmental treatment, and biomedical and sensing applications [3, 5]. Hence, the utmost technology used for the treatment of organic wastewater and related applications is attributed to the evolving solar light-driven photocatalysts [3].

Semiconductor photocatalysts can be used for the removal of ambient concentrations of organic and inorganic species from aqueous or gas phase systems in drinking water treatment, environmental tidying, and industrial and health applications. This is due to the massive ability of these semiconductors (g-C_3_N_4,_ TiO_2_- and ZnO) to oxidize organic and inorganic substrates in air and water through redox processes for its effective application in solar energy utilization, wastewater, and environmental treatment, biomedical and sensing applications without any second pollution.

Polymeric graphitic carbon nitride (g-C_3_N_4_) has become the prime center for consideration in photocatalysis research [6]. g-C_3_N_4_ is a visible-light-response element with band gap of 2.7 eV, and the energy location of CB and VB is at − 1.1 and 1.6 eV via normal hydrogen electrode respectively [Wang et al. 2009]. In addition, g-C_3_N_4_ has the ability to resist attacks from heat, strong acid, and strong alkaline solution [7]. g-C_3_N_4_ has a unique ability to be simply prepared by thermally polycondensing the cheap N-rich precursors, such as dicyanamide, cyanamide, melamine, melamine cyanurate, and urea, and this is unlike the other metal-containing photocatalysts that require costly metal salts for preparation [6, 8]. Thermal condensation, solvothermal, chemical vapor deposition, microwave-assisted, polymerization, and hydrothermal synthesis are examples of preparative strategies (Table 2) which have been commendably applied in the preparation of carbon nitride for distinctive purposes and analysis in the area of photocatalysis and others [9].

Due to these outstanding properties of g-C_3_N_4_, the use of this promising g-C_3_N_4_ in water splitting, CO_2_ photo reduction, organic contaminants purification, catalytic organic synthesis, and fuel cells is more efficient and effective [6]. The number of admirable researches and reviews on g-C_3_N_4_ structure and preparation in the last few years has increased tremendously [10]. Authors mainly laid emphasis on the most contemporary advances on the structure, synthesis, and preparation techniques of g-C_3_N_4_ and carbon nitride (CN_x_) films vividly in this concise mini review. The unique structure and the novel synthesis and preparation techniques of g-C_3_N_4_, and CN_X_ films are nicely presented, and the enlightened concepts on extending the preparation of g-C_3_N_4_ in this mini review are then emphasized. Also, the authors discussed the applications on g-C_3_N_4_, and the perspectives in future researches were also advocated.

## Review

### Graphitic Carbon Nitride and Photocatalysis

**Fig. 1:**
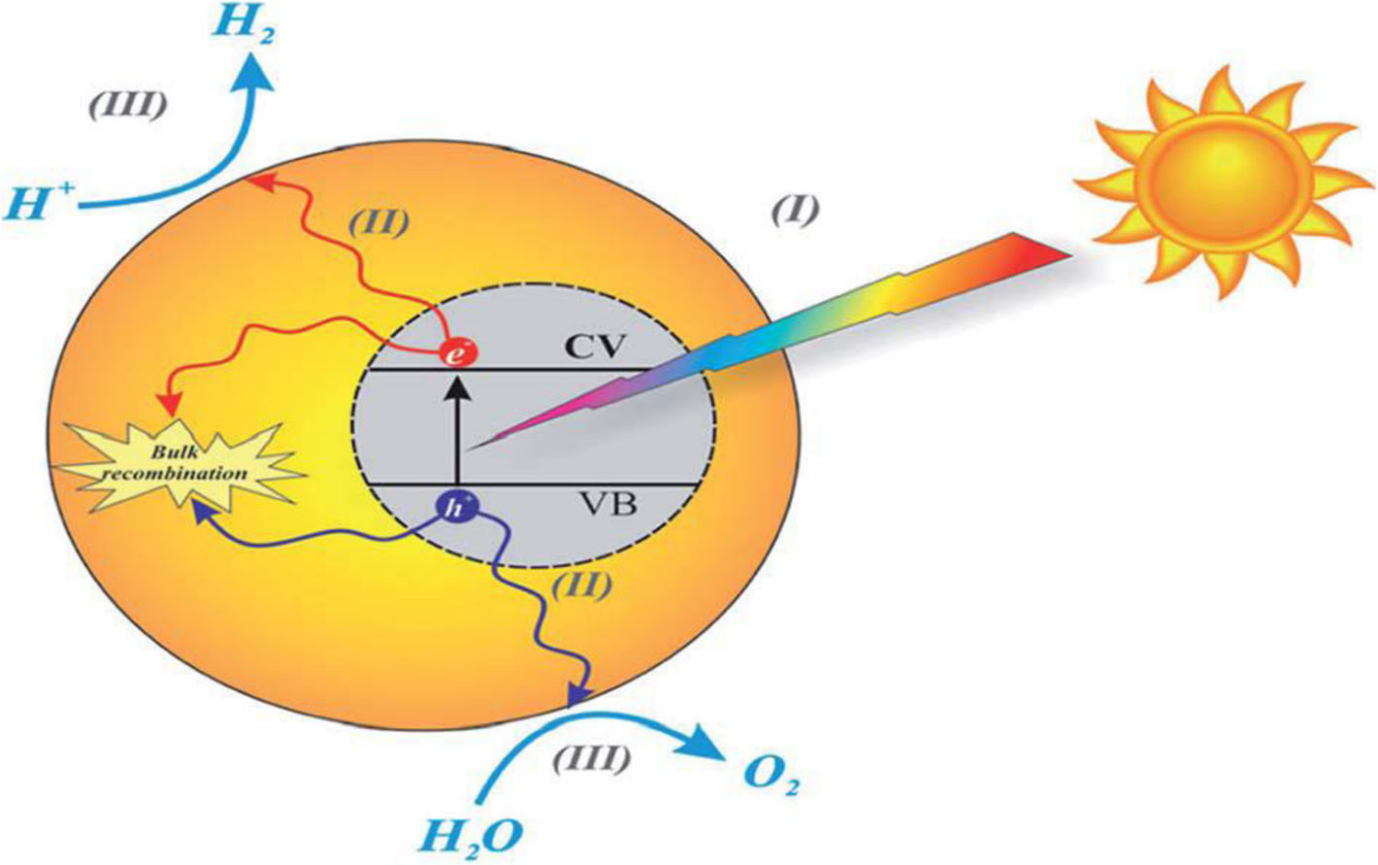
Schematic diagram of the basic mechanisms of the photocatalytic activity of water splitting. Reproduced with permission [113, 114]. Copyright 2015 & 2018. The Royal Society of Chemistry

**Fig. 2:**
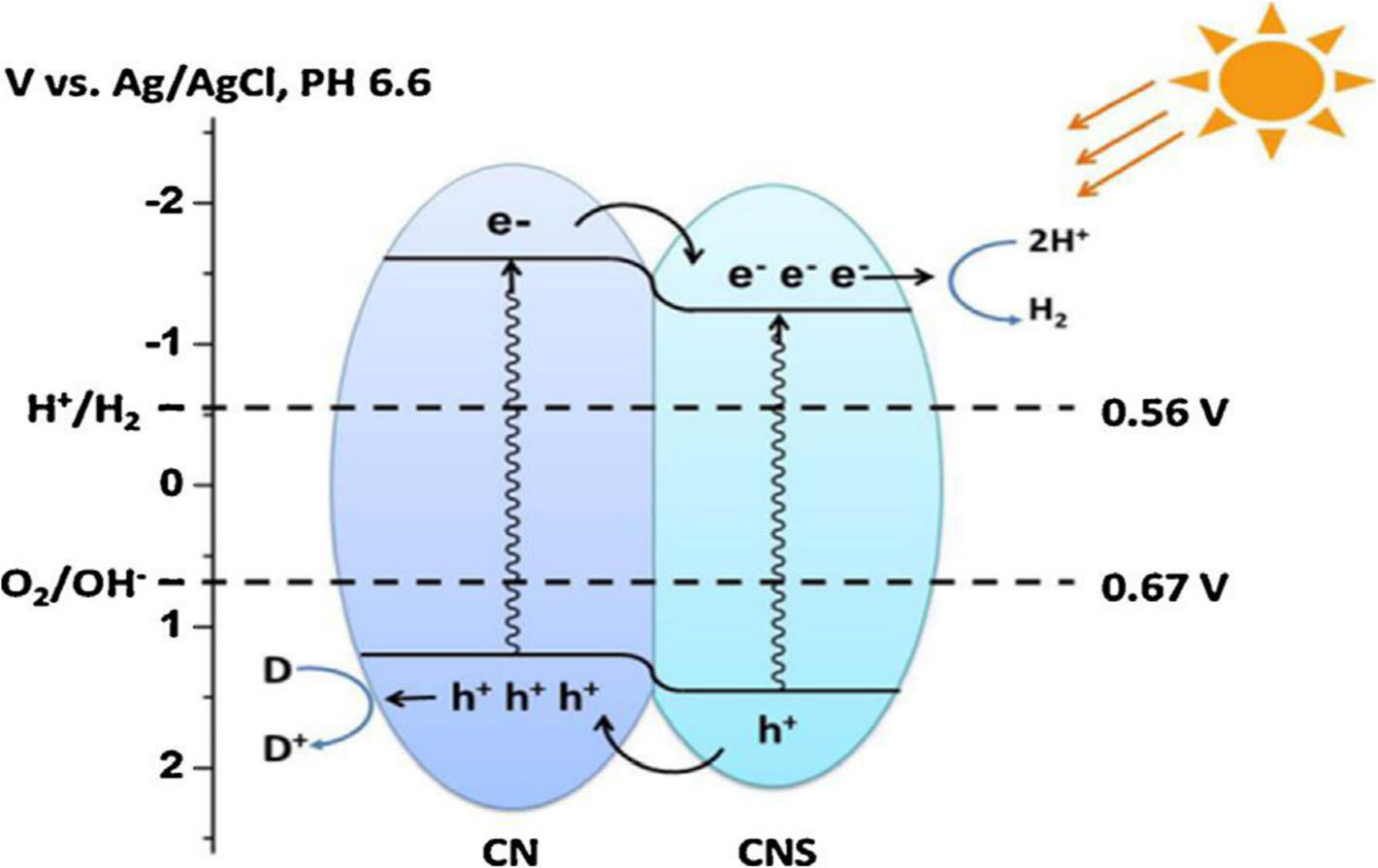
Schematic illustration of organic heterojunction formed between g-C3N4 and S-doped g-C3N4. Reproduced from Ref. [115]. Copyright 2015. Elsevier

Photocatalysis is best referred to the acceleration of chemical conversions (oxidations and reductions) brought about through the activation of a catalyst. This reaction involves a semiconductor either alone or in combination with metal/organic/organometallic promoters, through light absorption, following charge or energy transfer to be adsorbed which can lead to the photocatalytic transformation of a pollutant. During a photocatalysis mechanism, there is a simultaneous occurrence of at least two main actions which aids a successful production of reactive oxidizing species (Fig. 2). These reactions are oxidation of dissociatively adsorbed H_2_O mostly generated by photogenerated holes and reduction of an electron acceptor also created by photoexcited electrons (Fig. 2). Hence, these reactions produce a hydroxyl and superoxide radical anion, respectively [11]. During photocatalysis reaction, it is obvious that there is photon-assisted generation of catalytically active species instead of the action of light as a catalyst in a reaction [12–15, 16]. Considerably, reaping of visible light, mostly from sunlight, by catalyst (photocatalyst) to initiate chemical transformations (Fig. 1) is described as photocatalysis. Application of C_3_N_4_ photocatalyst for wastewater treatment, solar energy utilization, environmental treatment, and biomedical and sensing applications has been discussed in many areas of science.

Enlightenment of a semiconductor catalyst, such as TiO_2_, ZnO, ZrO_2_, and CeO_2_, with photons carrying energy equal or in excess of its band gap, creating an electron hole pair similar to photo-induced electron transfer and absorption of light promotes one electron into the conduction band. The oxide may transfer its electron (Fig. 2) to any adsorbed electron acceptor (thereby promoting its reduction), while the hole (or the electron vacancy) may accept an electron from an adsorbed donor (promoting its oxidation). g-C_3_N_4_ is capable of catalyzing hydrogen/oxygen evolution and CO_2_ reduction under band gap excitation and in the presence of suitable co catalysts and/or sacrificial agents.

### Graphitic Carbon Nitride Nano-Based Particle

Materials with 1D nanostructures having distinct electronic, chemical, and optical properties could have their size and morphology adjusted. This ability of the 1D nanostructured materials has led to a novel advancement of diverse approaches to improve their photocatalytic activity [17]. In addition, there is guidance of electron movement in the axial direction and lateral confinement of electrons by these 1D nanostructures. There has been advancement of 2D materials from graphene to metal oxide and metal chalcogenide nanosheets and then to 2D covalent organic frameworks (g-C3N4).

**Fig. 3:**
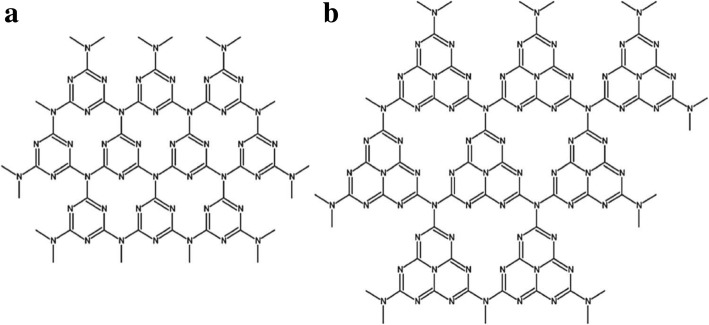
**a** Tri-s-triazine and **b** tri-s- triazine as unit structures of g-C_3_N_4_. Reproduced with permission [25, 31]. Copyright 2008 Royal Society of Chemistry

The appropriate means of selection of precursors and condensation methods had led to two main types of g-C_3_N_4_ structural polymorphs and this includes, firstly, the g-C_3_N_4_ consist of a condensed s-triazine units (ring of C_3_N_3_) with a periodic array of single-carbon vacancies. The second type of g-C_3_N_4_ consists of the condensed tri-s-triazine (tri-ring of C_6_N_7_) subunits coupled through planar tertiary amino groups, and this has greater periodic vacancies in the lattice. The g-C_3_N_4_ networks mainly consists of melon-based segments (the second type structure; this consists of the tri-s-triazine unit, Fig. 3a) which is thermodynamically more stable compared to the melamine-based arrangements (the first type structure; this compose of the s-triazine, Fig. 3b) as described by the functional theory (DFT) calculations [18]. Hence, it is broadly believed that the tri-s-triazine nucleus is the fundamental building blocks for the formation of the g-C_3_N_4_ network.

#### Structure of Graphitic Carbon Nitride Nano-Based Particle

g-C_3_N_4_ are a class of two-dimensional (2D) polymeric materials comprising entirely of covalently linked, sp_2_-hybridized carbon and nitrogen atoms. Carbon and nitrogen have the distinction of various valence states forming bonding; therefore, in g-C_3_N_4_, there are diverse of valence bond structures. Research works have initiated that some C_3_N_4_ defect structures and amorphous structures of g-C_3_N_4_ are still the metastable structures, but with the upturn of N vacancy, these two kinds of configuration of g-C_3_N_4_ material usually lessen in bulk modulus. The structural characteristics, composition of materials, and crystallinity of g-C_3_N_4_ can be characterized and evaluated by XRD, XPS, and Raman techniques. In 1830, Berzelius described the general formula (C_3_N_3_H) n and Liebig also devised the notation “melon,” and these predictions had then led to more research focused on carbon nitride oligomers and polymers [19, 20]. Furthermore, these crystal structures have been found and stated in experiments [21–23]. The α-C_3_N_4_ is earlier found by Yu and coworkers [24]. A graphite-like loaded 2D structure of the graphite C_3_N_4_ is usually observed as nitrogen heteroatom- substituted graphite framework which mainly includes p-conjugated graphitic planes, and it is with sp^2^ hybridization of carbon and nitrogen atoms. Crystalline graphite is 3% less dense than the g-C_3_N_4_. Shifting the localization of electrons and then consolidating the bonds that is between the layers due to the nitrogen heteroatom substitution can help enlighten the interlayer distance of g-C_3_N_4_ [25].

#### Electronic Structure and Properties of g-C_3_N_4_

Currently, g-C3N4 is considered as a new-generation photocatalyst to recover the photocatalytic activity of traditional photocatalysts like TiO_2_, ZnO, and WO3. g-C3N4 is assumed to have a graphitic-like structure [26–28, 29, 30]. Thermal polycondensation method is generally used to prepare g-C3N4 and, hence, to investigate the electronic structure of g-C3N4.

The α-C_3_N_4_ is earlier found by Yu and coworkers [24]. These scientists used the calculation procedure of quantum mechanics clusters model and developed α-C_3_N_4_ by optimization the electronic structure of g-C_3_N_4_ for photocatalysis and others. In the structure of alpha-C_3_N_4_, C and N atoms linked by sp^3^ key was to used design the tetrahedron structure of g-C3N4. Liu and Cohen anticipated the existence of beta-C_3_N_4_ by means of band concept of first principles and prepared beta-C_3_N_4_ based on *β*-Si_3_N_4_ electronic structure. Liu and Cohen then revealed that the structure of *β*-C_3_N_4_ was hexagonal encompassing 14 atoms for each unit cell.

The outstanding prediction anticipated by Liu and Cohen in 1989 that the b-polymorph C_3_N_4_ would have exceptional high hardness values in comparison with diamond has enthused scientific research to date [26]. In 1993, C_3_N_4_ thin films via magnetron snorting of a graphite target on Si (100) and polycrystalline Zr substrates under a pure nitrogen ambience and consideration of the structure of C_3_N_4_ with analytical electron microscopy and Raman spectroscopy were synthesized by Chen and co-authors [27, 31]. Scientists, Teter and Hemley [28], foretold that alpha-C_3_N_4_, beta-C_3_N_4_, cubic-C_3_N_4_, pseudo cubic-C_3_N_4_, and graphite C_3_N_4_ show pronounced hardness approaching that of diamond in their experiment which they performed 3 years later as already described in accordance with first-principle calculations of the relative stability, structure, and physical properties of carbon nitride polymorphs.

Wang and coworkers [26, 32] applied ab initio evolutionary algorithm structure searches to calculate the precise structure of g-C3N4 prepared by thermal polycondensation and salt-melt synthesis methods for an enhanced visible-light-responsive photocatalysis. The most stable structure 1–3 were predicted for heptazine-based g-C3N4. The order of phase stability was 1 > 2 > 3. Contrary to other layered structures, distorted phases in heptazine-based g-C3N4 (see Fig. 3) were the most stable. This structure contributes the enhanced photocatalytic activity of the promise. In g-C3N4, lone pair electrons of nitrogen are mostly accountable for band structure and development of valence band.

### Preparation of Graphitic Carbon Nitride Nano-Based Particle

#### Synthesis

The interesting tribological and electronic nature of graphitic carbon nitrides makes it possible to develop a method to deposit layers of graphitic carbon nitrides in a controlled manner; hence, graphene nitride can be obtained. Considerably, the benchmark particle for comparison is the bulky g-C3N4. This particle can be best achieved by using selection of nitrogen-rich precursors without direct C–C bonding, such as cyanamide, dicyandiamide, melamine, thiourea, urea, or mixtures through various preparative methods (Tables 1, 2, and 3), for instant, thermal condensation [33]. Carbon nitride materials are mostly bulk resources with small surface area, usually less than 10 m^2^ g^−1^ when they are prepared or synthesized by direct condensation of the nitrogen-containing organic precursors [34].

**Table 1:** Comparisons between hard templating and soft templating approaches used for g-C_3_N_4_ synthesis

Fabrication strategies	Comparisons	References
1. Hard templating approach	i. The nano-casting technique using a hard template is the most widely reported and successfully applied method used for the introduction of mesoporosity in solid materials such as carbons, nitrides, polymers, and ceramics. ii. Nano and casting differ in terms of the length scale involved. While casting is predominantly done on a macroscopic scale, nano-casting on the other hand is done on the nanoscale, and hence, the prefix “nano” is used while referring to the casting process as applied to the synthesis of materials with nano dimensions. iii. This synthetic method involves the following three important steps: (a) synthesis of the ordered mesophase silica template; (b) infiltration of the template with the necessary precursors, including the conversion of precursors into a solid; and (c) removal of the template.	[116, 117]
2. Soft templating approach	i. A soft templating approach has been extensively used for the synthesis of many mesoporous materials. ii. Unlike the broadly reported hard template-based nano-casting procedures for the synthesis of graphene carbon nitride reports, on the use of soft templates for the synthesis of graphene are quite limited. iii. The soft templating approach was appreciated by Antonietti and group for the preparation of carbon nitride through simple self-assembly between the organic structure directing agents and the CA.	(Fig. 4a), [25, 118, 119]

**Table 2:** Typical g-C_3_N_4_ preparation techniques

Methods	Precursors	Surface area	Photocatalytic activity	Sharpe/structure	References
Thermal reactions	Melamine, cyanuric chloride	No data	High	Fine nickel powder	[41]
Solvothermal reactions	Melamine, cyanuric chloride, urea	No data	High (Fig.7)	Crystalline, fine particles (Fig.7)	[59, 60, 63]
Chemical vapor deposition	Melamine, uric acid	Large	No data	Heptazine blocks, jaggy-like shape (Fig. 8), crystallinity, nanometric texture	[60, 66]
Sol–gel synthesis	Dialkylamine	Higher	No data		[69, 70]
Microwave heating	Melamine, cyanuric chloride, urea	high (90 m^2^ g^−1^)	Enhanced	No data	[71]

**Table 3:** Comparisons of some selected Fabricating strategies of g-C_3_N_4_ synthesis

Techniques	Comparisons		References
	Characteristics	Previous studies	
1. Supramolecular pre-assembly	a. Molecules adopt a well-defined arrangement into stable aggregates by non-covalent bonds under equilibrium conditions b. Hydrogen bonding is highly essential in arranging the structure of supramolecular aggregates due to the directionality and specificity of this kind of inter-actions. c. The precursor melamine can link with triazine derivatives, such as cyanuric acid, into supramolecular aggregates through hydrogen bonds	a. The use of melamine–cyanuric acid (CM) complex as starting materials was reported by Thomas and coworkers and Antonietti and coworkers. It was found that the CM morphologies depend on the used solvent for melamine–cyanuric acid molecular assembly, leading to various well-organized g-C_3_N_4_ with different morphologies	[6, 21, 26–28]
2. Molten salt strategy	a. Salt-melt synthesis usually acts as a solvent for high-temperature materials synthesis including many organic and inorganic reactions	a. Zou et al. successfully synthesized a carbon nitride intercalation compound by heating the melamine with a low melting point eutectic mixed salts under air and ambient pressure. Interestingly, g-C_3_N_4_ nanotubes were produced. The resultant g-C_3_N_4_ nanotubes are very stable and active for solar H_2_ production (Fig. 8b).	(Fig. 8a), [41, 98]
3. Ionic strategy	a. This strategy possesses high chemical and thermal stability, small vapor pressure, and the liquid nature at ambient. b. Makes ionic liquid to be used as solvents in many fields	a. Reported the usage of 1-butyl-3 methylimidazolium tetrafluoroborate (BmimBF4) ambient ionic liquid as soft template and dicyandiamide (DCDA) as precursor to synthesize the boron- and fluorine-containing mesoporous-g-C_3_N_4_. Very interestingly, no micropores are present in obtained g-C_3_N_4_	[32]

Mesoporous structure, when mineralized and the specific surface area amplified, helps to fine-tune the physicochemical properties and then increases the photocatalytic performance of graphite carbon nitride (g-C_3_N_4_). Nano-casting/replication of mesoporous silica matrices is the first method used to prepare graphite carbon nitride (g-C_3_N_4_), these were famous for their cohort of the corresponding carbon nanostructures [35]. Great efforts were then put in place to bring out more innovative schemes for g-C_3_N_4_ modification, which was enthused by the hard template method (Table 1). Liu and Cohen then discovered the (Table 1) soft template technique [26], and the other g-C_3_N_4_ modification schemes such as acidic solution impregnation, the ultrasonic dispersion method, and chemical functionalization [26] were also discovered. These methods as described above were good signs of the principle in modifying the surface chemical properties and the texture of g-C_3_N_4_, alone with its electronic potentials.

Thermal treatments, such as physical vapor deposition (PVD) [36], chemical vapor deposition (CVD) [37], solvothermal method [38], and solid-state reaction [38], are used for polymerizing plentiful nitrogen-rich and oxygen-free compound precursors comprising pre-bonded C–N core structures (triazine and heptazine derivatives), and these serve as the basic techniques for graphite carbon nitride (g-C_3_N_4_) synthesis. The commonly used precursors for the preparation of graphite carbon nitride (g-C_3_N_4_) through polymerization include cyanamide [39], dicyandiamide [40], melamine [41], urea [42], thiourea [43], guanidinium chloride [44], and guanidine thiocyanate [45]. The use of accomplished elements directly is actually challenging in many areas; this is due to the weak dispersity and ordinary nature of the bulk g-C_3_N_4_. The use of ample micro/nanostructures and morphologies to prepare different kinds of g-C_3_N_4_ has been intensely researched by scientist over the few last years of photocatalysis studies. For example, ultrathin g-C_3_N_4_ nanosheets which were prepared by exfoliating bulk g-C_3_N_4_ materials [46–48] were negatively charged and could be well dispersed in water.

**Fig. 4:**
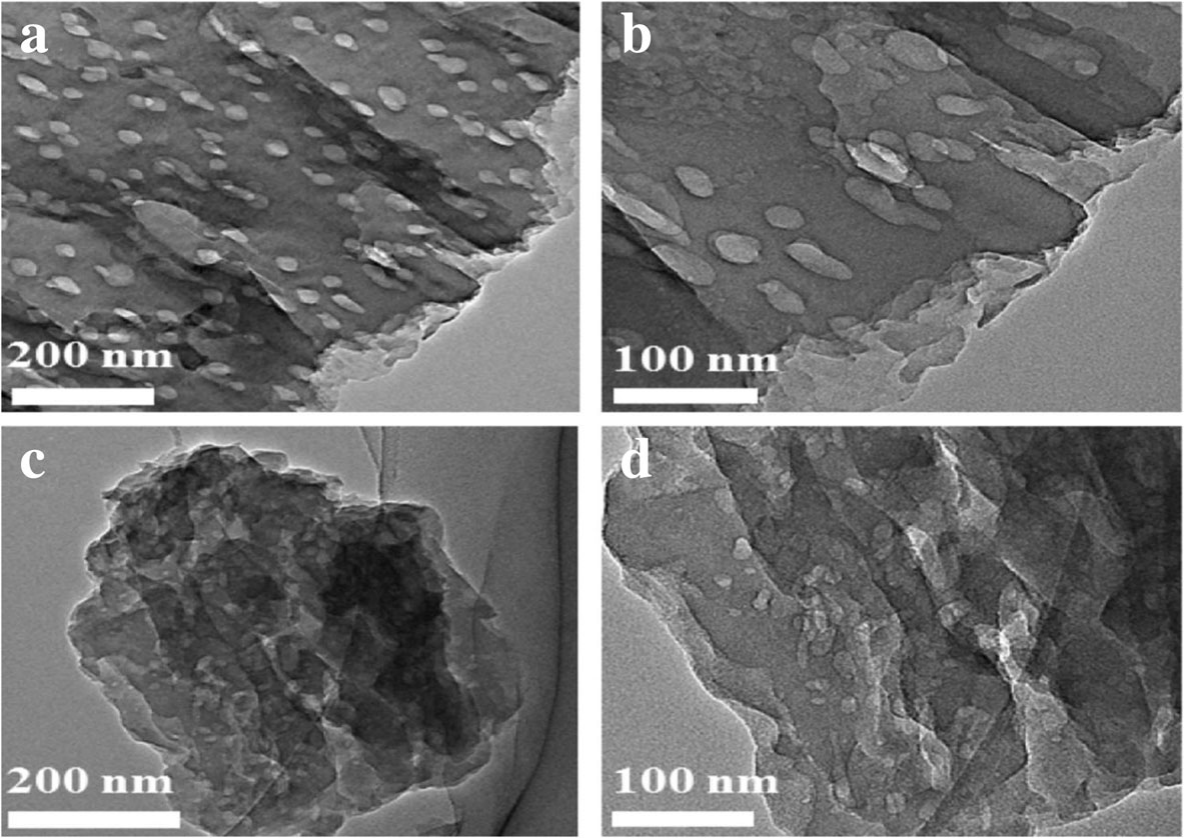
TEM images of TCN (**a** and **b**) and MCN (**c** and **d**) using a hard templating approach. Reproduced with permission from [120]. Copyright 2015. Elsevier

**Fig. 5:**
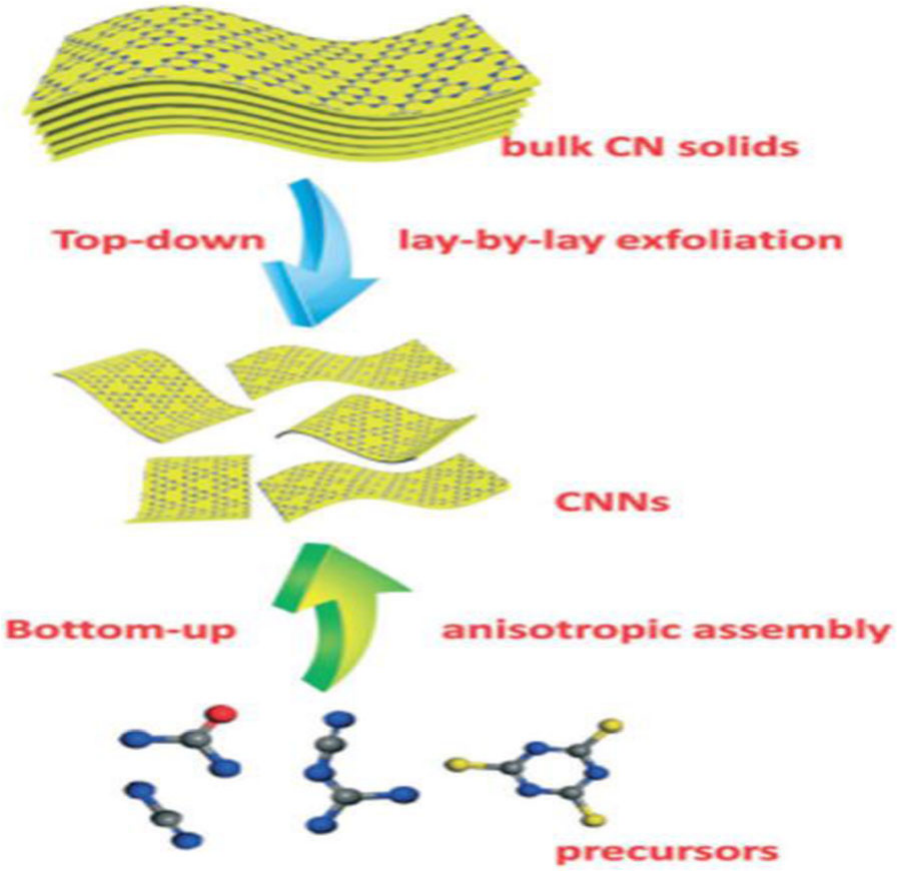
Schematic illustration of synthesizing CNNs by using the top–down and bottom–up strategies (reproduced from ref. [121] with permission from The Royal Society of Chemistry)

**Fig. 6:**
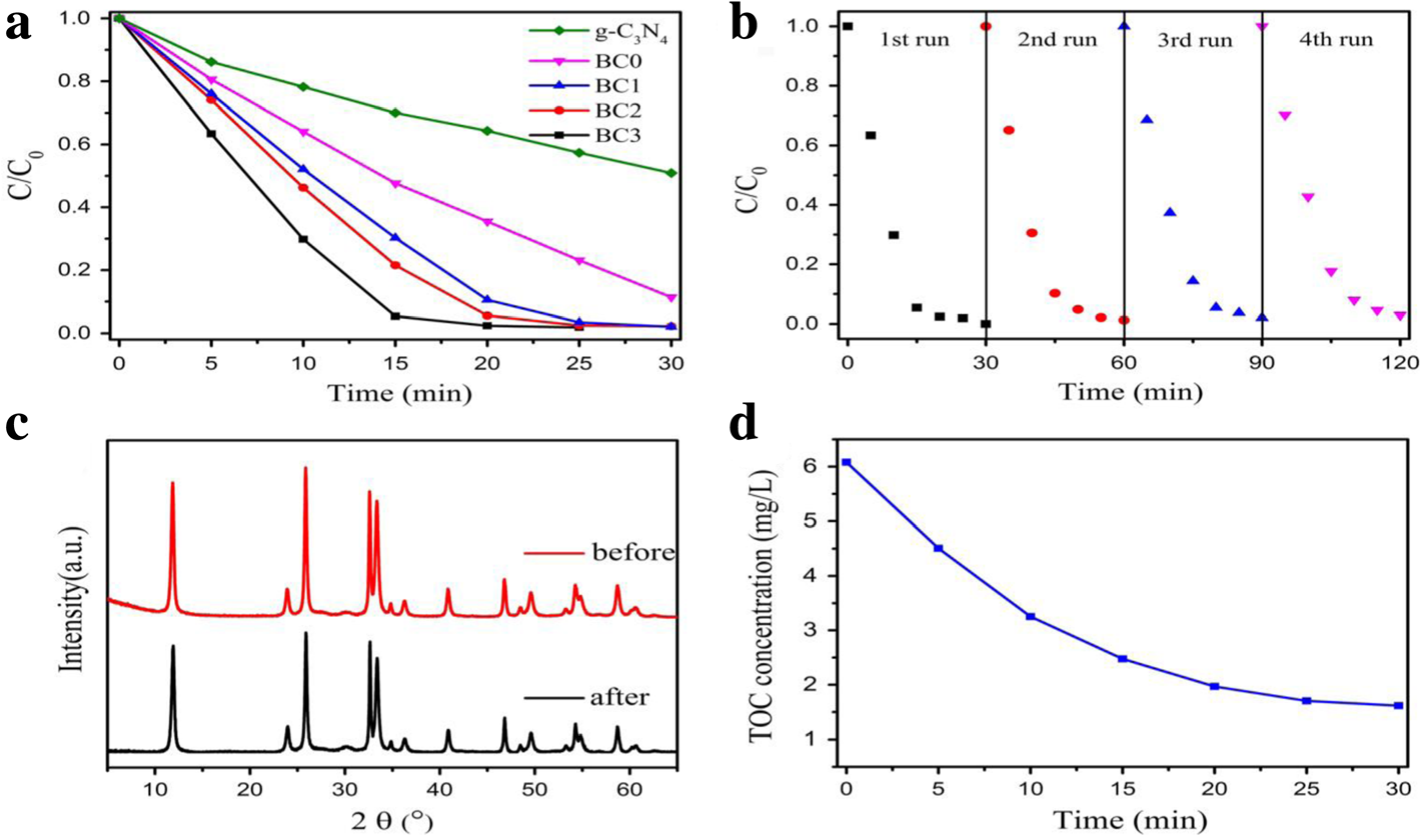
Schematic diagram of Preparation and Enhanced Visible-light Photocatalytic by the decrease of RhB by different photocatalysts as a function of visible light irradiation time (photocatalysts loading, 0.5 g/L; initial RhB concentration, about 10 mg/L, without pH modulation). The photocatalysts used were pure g-C3N4 and **a** series of g-C3N4/ BiOCl hybrids, **b** cyclic degradation of RhB over BC3, **c** XRD patterns of BC3 photocatalysts before and after the photocatalytic process, and **d** plots of TOC versus degradation time. (Reproduced from ref. [122] with permission from Springer-Verlag GmbH Germany 2017)

Thermal oxidation exfoliation, ultrasonic exfoliation, and chemical exfoliation are well known as the major exfoliation methods used for preparing g-C_3_N_4_ materials. Meso-g-C_3_N_4_ materials have great performances such as great photocatalytic activity due to their greater specific surface area (up to 830 m^2^ g^−1^) and larger porosity (up to 1.25 cm^3^ g^−1^); also, the larger numbers of active sites present on the surface and higher size or shape selectivity enhances their excellent performances. The utmost essential pathways for the preparation of meso-g-C_3_N_4_ include soft templating (self-assembly) [49, 50] and hard templating (nano-casting) [51] methods (Table 1 and Fig. 4). Smaller sizes, popularly known as g-C_3_N_4_ quantum dots (QDs), were used by a lot of great research scientists in their researches for the synthesis of g-C_3_N_4_ [52–55]. Two main approaches to synthesize 2D g-C_3_N_4_ nanosheets are delamination of layered g-C_3_N_4_ solids into free-standing nanosheets mostly known as top–down strategy (Fig. 5) and the anisotropic assembly of organic molecules in a 2D manner (Fig. 6), also called bottom–up strategy. [56] Remarkably for the diverse chemical structure and electronic band structure of the CN nanosheets, the as-prepared CN nanosheets revealed a unique electrochemiluminescence (ECL) emission response to numerous metal-ions. Due to this, there has been a successful development of ECL sensor with rapid detection of numerous metal-ions.

#### Techniques Used in Preparing Graphitic Carbon Nitride Nano-Based Particle

The study on the syntheses of carbon nitride (g-C_3_N_4_ and CN*x*) has enthused the curiosity of researchers from all over the world. g-C_3_N_4_ and films with precise photocatalytic properties have been synthesized [57, 58]. Thermal condensation, solvothermal, chemical vapor deposition, microwave-assisted, polymerization, and hydrothermal synthesis approached are methods (Table 2) which have been effectively used in the preparation of carbon nitride for different purposes and analysis in the area of photocatalysis and others [9].

##### Thermal and Solvothermal Treatment Methods

**Fig. 7:**
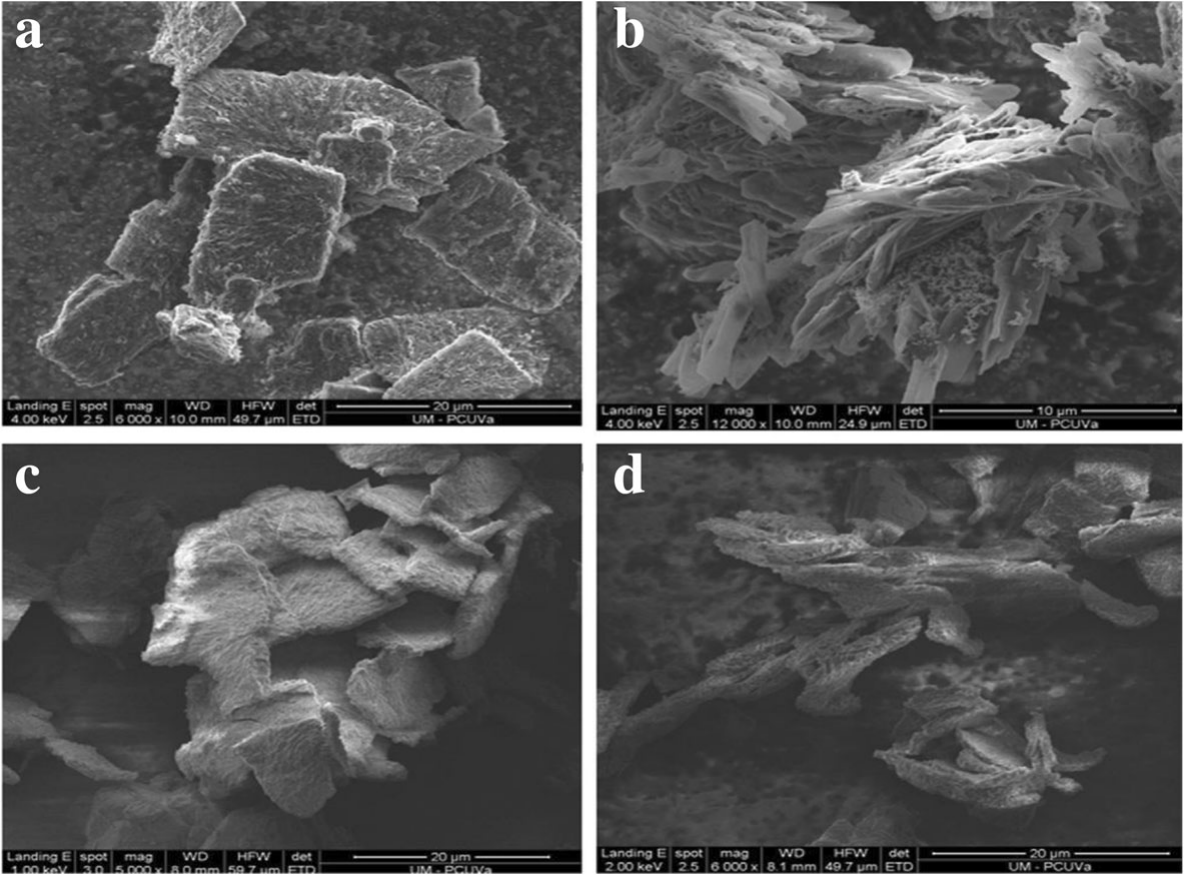
SEM images of sample B: (**a**) alumina particles coated with carbon nitride; (**b**) detail of the projecting indentations of carbon nitride. It is possible to observe the jaggy shape of the carbon nitride sheets obtained by pyrolysis. SEM images of sample A: (**c**) and (**d**) views of alumina particles coated with carbon nitride. Reproduced from [60]

**Fig. 8:**
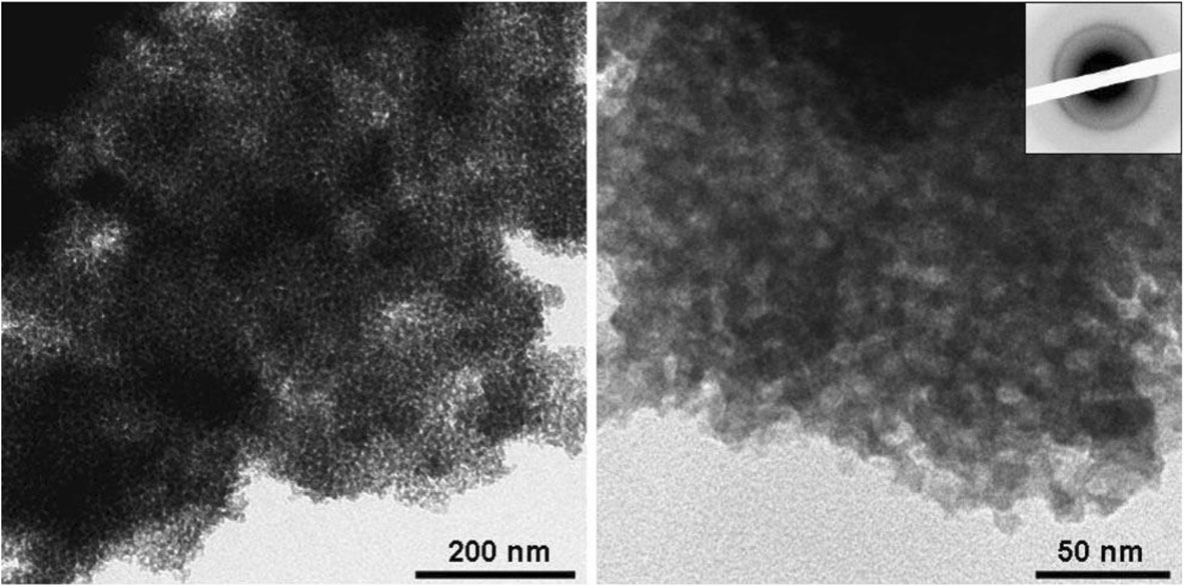
TEM images and an electron diffraction pattern of mp-C3N4 after removal of the silica nanoparticles. Reproduced with permission [123]. Copyright John Wiley & Sons Inc., 2006

Based on polycondensation reaction between melamine and cyanuric chloride in the presence of nickel powder, Li and research team [41] proposed two major methods for the synthesis of nitrogen-rich graphitic carbon nitrides. These two methods were solvothermal methods using benzene as solvent and solvent-free solid reaction way with thermal treatment (Fig. 7). Other works by many scientists [59–62, 63] suggested that solvothermal reactions usually produce crystalline after washing and drying, and do not require post annealing treatment. These scientists also proposed enhanced photocatalytic activities with this method (Fig. 8).

Niu and co. also reported the morphological changes when solvothermal technique was used [64]. Loumagne and coworkers [65] testified the physicochemical possessions of SiC-based deposits, achieved via the thermal decomposition of CH3SiCl3 in hydrogen. Kelly and group [66] reported synthesis of TaC by using reactants tantalum (V) chloride and carbon mixed under an argon-filled glove box through the thermal process. Successively, thermal condensation method, which mostly consists of conjugated aromatic heptazine system with graphitic assembling characteristics, has been used several moments to prepare g-C_3_N_4_ [36]. The use of solvothermal technique for g-C_3_N_4_ synthesis has great remunerations such as even and fine particle formation, little energy consumption, and higher economic feasibility as compared to the outdated thermal condensation method. Conversely, these methods are still time-consuming, demanding to a certain extent of a few hours to complete particle formation and crystallization.

##### Chemical Vapor Deposition

Investigation by Roberto and coworkers [60] suggested the use of chemical vapor deposition (CVD) for graphitic carbon nitride synthesis by the reaction between melamine and uric acid has high photocatalytic activity. It was found that the formed graphitic carbon nitride was with a structure based on heptazine blocks.

Roberto and coworkers then proposed that these carbon nitrides’ nature revealed a jaggy-like shape (Fig. 7), crystallinity, and a nanometric texture. Kelly et al. [66] has reported the synthesis of TaC by using reactants tantalum (V) chloride and carbon mixed under an argon-filled glove box via thermal technique and later transformed to TaC nanoparticles via chemical technique. CVD is one of the most useful methods to prepare monolayer graphene of high structural quality for use in different devices for catalytic activities [67]. Wang and group [26, 32] obtained CN푥 films on Ni substrate by using HFCVD method firstly. Because the preparation of these films is more likely to produce C–H and N–H linkage under the CVD conditions, most of the CN푥 films are amorphous. From previous studies, CVD procedures are used to prepare carbon nitrides, the choice of substrate materials is very critical to be considered. Large area samples can be prepared by exposing a metal to different hydrocarbon precursors at high temperatures. There are different types of CVD methods available such as plasma-enhanced CVD, thermal CVD, and hot/cold wall CVD. CVD methods mainly consist of electron cyclotron resonance, hot filament-assisted, DC glow discharge, radiofrequency discharge, and microwave plasma chemical vapor deposition. Bias of auxiliary hot filament chemical vapor deposition (HFCVD) is one of the local tools used in the deposition of diamond films and others. The exact mechanism of the formation of graphene depends on the growth substrate but typically initiates with the growth of carbon atoms that nucleate on the metal after decomposition of the hydrocarbons, and the nuclei grow then into large domains [68]. Recently, produced high-quality monolayer graphene by using resistive heating cold wall CVD was also 100 times faster than conventional CVD.

##### Sol–Gel Synthesis

Sol–gel synthetic technique is a process through which a solid product or a nano-material is formed from a solution after the transformation of the gel intermediate. In this synthesis method, reactants are mixed at the molecular level allowing fast reactions and lead to more homogeneous products with higher surface area. Remarkably, this technique has been used to synthesize different types of nanoparticles including metal carbide, and nitride processes for photocatalysis [69]. The synthesis of metal nitride using sol–gel processes can be traced back to the use of metal-organic compounds (synthesized from metal element and dialkylamine) [70].

##### Microwave Heating

**Fig. 9:**
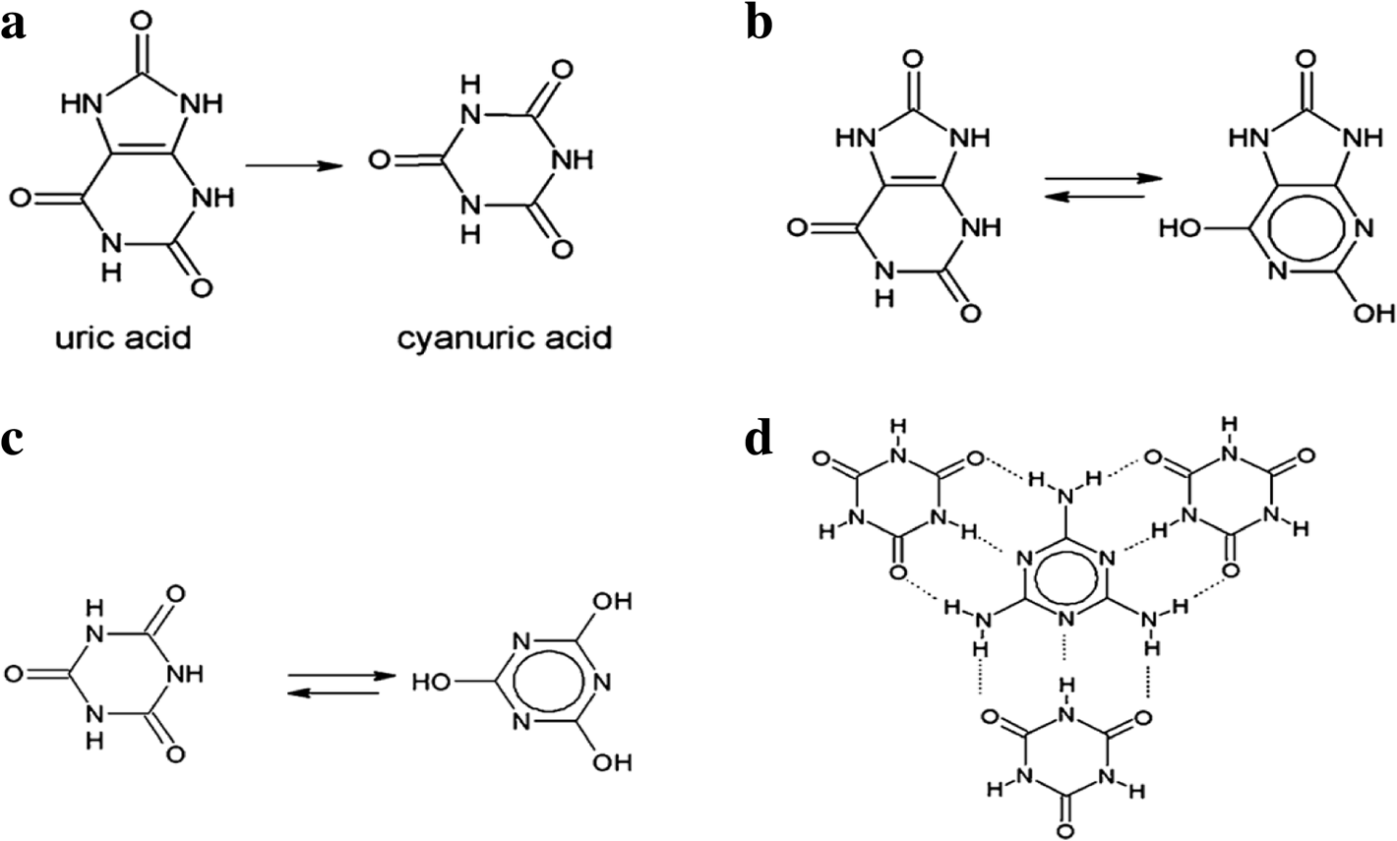
(**a**) Thermal decomposition of uric acid to cyanuric acid; (**b**) tautomers of uric acid; (**c**) tautomers of cyanuric acid; (**d**) schematic representation of a layer fragment of the adduct called melamine cyanurate

In recent times, microwave heating has been used widely for the preparation of fine chemicals and pharmaceuticals as compared to the methods described above, because it permits comprehensive reaction range and short reaction time, which are appropriate for production on an industrial scale [71]. A simple-minded technique was adopted by Wang and coworkers to synthesize g-C_3_N_4_ using a cheap/less-expensive nitrogen-rich precursor which can then be active as a photocatalyst for the generation of H_2_ and O_2_ under visible-light irradiation for their research. Microwave radiation speed up the chemical reaction and decrease the energy consumed, consequently penetrating the reaction vessel and openly making available energy to the reactants and solvent with a great heat transfer rate. Microwave heating technique is unlike traditional techniques such as oil baths and heating chambers; this method is more effective and reliable. Microwave radiation, regarding to heat solvothermally pressurized and closed reaction system, the reactants can be reacted and transformed into products far more swiftly than using the conventional method. Dai and coworkers proposed a time-saving and economical process for the synthesis of g-C_3_N_4_ using microwave-assisted polymerization recently. Dai and coworkers then found out that the g-C_3_N_4_ sample achieved, showing submicrospheres and a high surface area of 90 m^2^ g^−1^, (Fig. 9) and was successfully synthesized at 180 °C under microwave irradiation condition for only 30 min which revealed an enhanced photocatalytic performance [71]. Experiments performed by Hu and coworkers also revealed that the microwave- synthesized g-C_3_N_4_ has good chemical and thermal stability and strong emission intensity than those of the conventional one [71]. Hu and coworkers also stated that microwave synthesized g-C_3_N_4_ performed better in visible-light-responsive photocatalysis.

##### Physical Vapor Deposition

It consists of magnetron sputtering, ion beam deposition (IBD), reaction sputtering, and pulsed laser deposition, and so forth. Reaction sputtering is the elementary method for preparation of composites. When this technique is used to prepare g-C_3_N_4_, the mass fraction of nitrogen is usually less than 40%. Conversely, to form 훽-C_3_N_4_, the system should consist of an adequate amount of nitrogen and stoichiometric ratio should reach 57%. Niu and his group [72] achieved the g-C_3_N_4_ on silicon substrate by using pulse laser evaporation C target, auxiliary deposition of atom nitrogen. Niu et al. studies found that the amount of N reached 40% in the films and then C, N atoms combined with nonpolar covalent bond. Successively, Sharma et al. [73] and Zhang et al. [74] also did some critical studies and then obtained CN푥 films by a similar method as discussed. Mihailescu and coworkers [75] also used ammonia instead of N_2_-manufactured hard CN푥 films with carbon nitrogen single bond, double bond, and triple bond and then found out that its optical band gap is 4.5 eV. From the recent study, what scientists frequently get are mixture films which comprise several crystal phases.

To consider the efficacy of prepared g-C_3_N_4_, photocatalytic hydrogen evolution using crystalline carbon nitrides (CNs) was proposed by Takanabe and his group [76]. Takanabe et al. acquired carbon nitrides by supramolecular aggregation (Table 3) which was further monitored by (Table 3) ionic melt polycondensation (IMP) using melamine and 2, 4, 6-triaminopyrimidine as a dopant. There are other few methods similar to what Takanabe and his group used in their experiment, see Table 3.

### Applications of Graphitic Carbon Nitride

There are several emerging applications of this graphitic carbon nitride and such applications include based sensing, biomedical applications, wastewater and environmental treatment, solar energy utilization and being used in device making.

#### Solar energy Utilization

**Fig. 10:**
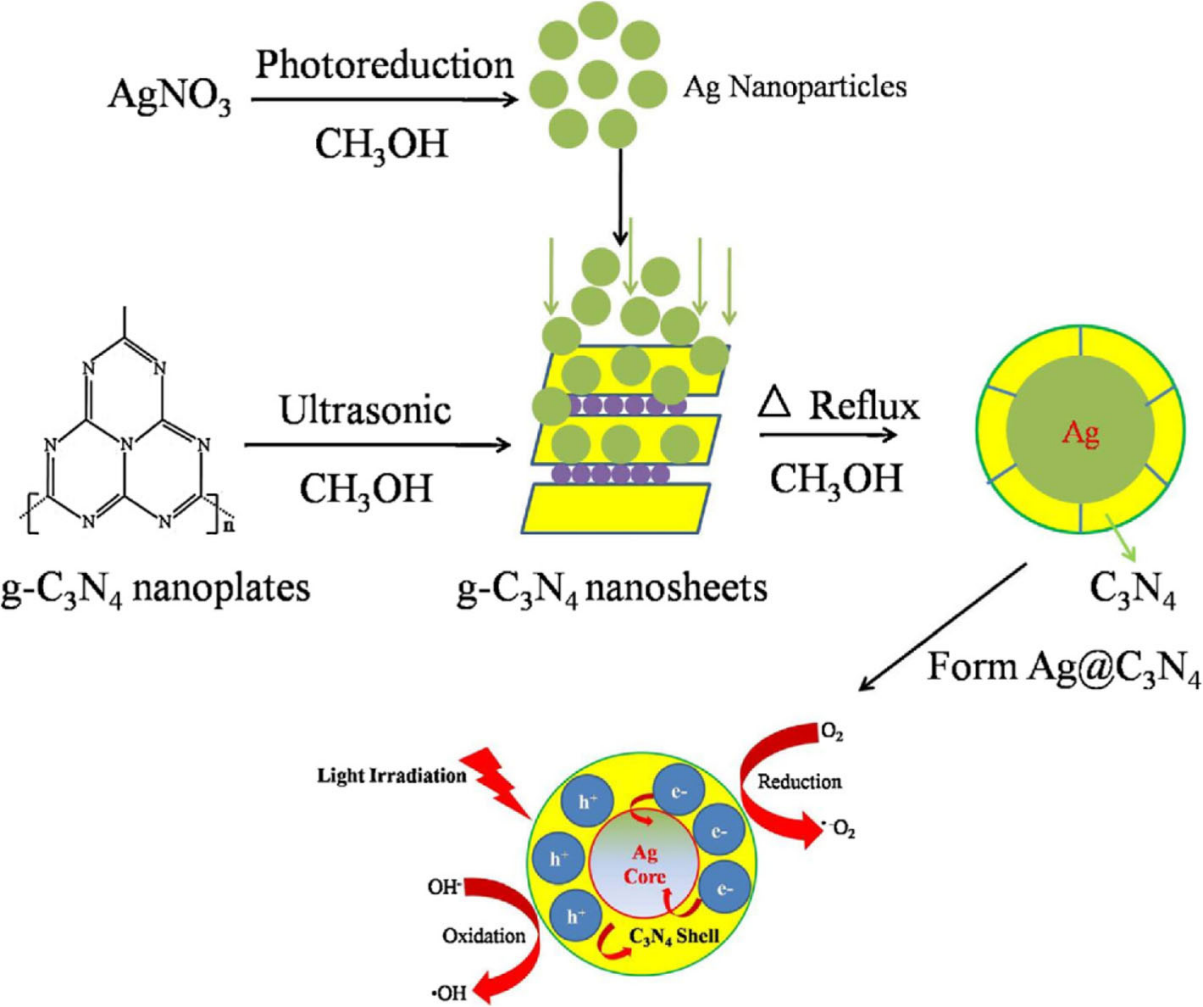
Schematic drawing illustrating synthetic route (templating method) and the mechanism of charge separation and photocatalytic process over C3N4 and Ag@C3N4 photocatalysts under light irradiation. Reproduced with permission [124]. Copyright 2014 Elsevier.

To increase the visible responsive activity of carbon nitride is not only dependent on controlling the molecule structures, synthesis, and preparation techniques of CN but also dependent on the ability to alter the electronic structures of these materials. Usually, under visible-light irradiations, carbon nitrides can be used to produce photoelectrode and thereby generating photocurrent. This ability of g-C_3_N_4_ is due to the exceptional reversible protonation and deprotonation nature. One of the greatest approaches is the use solar fuel from CO_2_ and water (produced by most photocatalysts) to produce H_2_, hydrocarbons, and syngas for energy and others [77, 78]. It was proposed that g-C_3_N_4_ has the potential of being metal-free and scalable photocatalysts for visible-light use based on the structure, synthesis, and preparation technique applied. A recent work by Liu and team [79] has suggested a novel development of sacrificial templating method for formulating mesoporous g-C_3_N_4_ spheres and a high-throughput scheme. This proposed technique can be used to synthesize g-C_3_N_4_ rods, and this is best for NADH regeneration (Fig. 10a–c) for successful production of energy and others.

#### Wastewater and Environmental Treatment

**Fig. 11:**
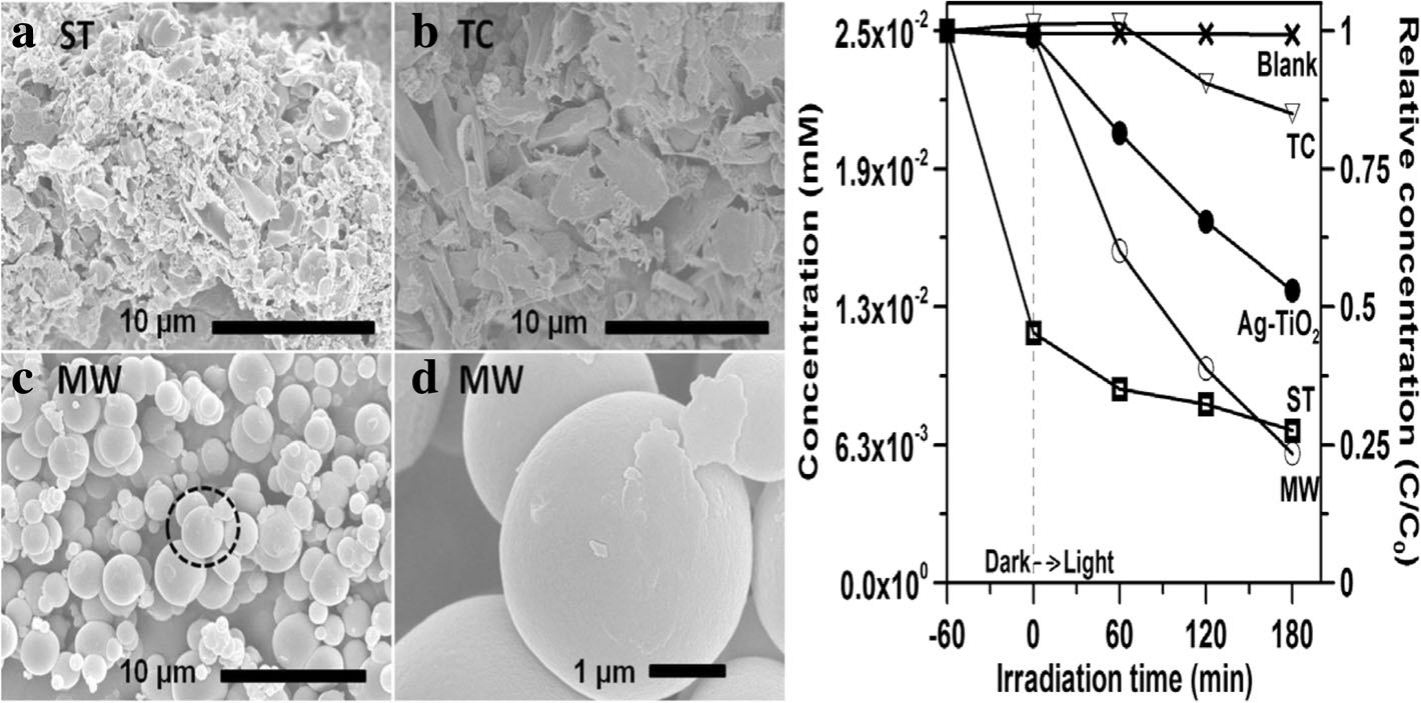
SEM images of (a) ST, (b) thermal condensation (TC), and (c) Microwave assisted synthesis (MW) samples; (d) magnification of MW sample; Photocatalytic degradation of MO solution over MW, ST, TC C3N4, and Ag-TiO2 samples irradiated under visible light. In the experiment, a blank test was performed in which the solution was irradiated without adding a catalyst. Reproduced with permission [125]. Copyright 2017 Elsevier

Most petrochemical, petrochemical, textile, and food industrial processes lead to pollution in the environment, to be precise, water bodies [80]. In the production of textiles, photographic materials, and printing materials, organic dyes are used and these dyes leach into most aquatic environment during the dying process [81]. Despite the harmful impact of these dyes on human and animal health, their biological and chemical degradation is challenging [82, 83]. Due this threat, there is a need to develop a superior oxidation process for the treatment of contaminated drinking water and non-degradable materials [84, 85]. Most researches [86–90] have proven that the use of semiconductors such as g-C_3_N_4_ for photocatalysis is the best method for the treatment of wastewater and environment due to their less harmful nature [86–90]. g-C_3_N_4_ is best known to be the potential photocatalysts for the degradation of numerous pollutants [16, 90, 91], with photophysical potentials of the parent nitride altered through doping with heteroatoms, heterojunction formation with other materials, and textural enhancements to expand surface area and porosity. The structure, synthesis, and preparation techniques of g-C_3_N_4_ nanosheets also determine the efficiency of the photocatalyst and its application in relation to wastewater treatment. Ultrathin g-C_3_N_4_ nanosheets derived from bulk g-C_3_N_4_ by exfoliation in methanol reveal heightened photocatalytic activity (Fig. 11) for methylene blue (MB) degradation [92]. Synthesizing and preparing of the candidate by doping metals such as Cu and Fe [93–95, 96] and non-metals such as B, C, O, or S [97–100], and co-doping [101–103] has been widely used by many scientists for water and environmental treatment. A promising solution to environmental depollution [104–106] is the combination of noble metals and g-C_3_N_4_ [107–112].

In summary, the unfeasible applications in wastewater and environmental pollution of most of the utmost well-versed photocatalysts is due to some of their demerit deterrents which includes, high cost, small scale, little photocatalytic activity, and thought-provoking recycle. Reasonably, in the area of environmental remediation, g-C_3_N_4_, TiO_2_-, and ZnO-based nano-material exhibit the most promising applications as result of their low cost, high photocatalytic activity, and no second pollution on the environment [3].

#### Biomedical and Sensing Applications

To increase the ability of g-C_3_N_4_ for sensing, biotherapy, and bioimaging usage, there is a need to alter the molecular structure, thereby enhancing the handling of the material in water. Due to the light photoluminenscence, highly recommended for biological related use, g-C_3_N_4_ nano-material is a very essential candidate for biomedical and sensing applications. The application of g-C_3_N_4_ for sensing, biotherapy, and bioimaging mainly considers its structure, synthesis, and preparative mechanisms. Zhang and coworkers [53] proposed that ultrathin g-C3N4 nanosheets could be used as biomarkers for the labeling of the cell’s membranes. g-C_3_N_4_ has also been suggested by Lin and co. to be a potential photosensitizers and pH-responsive drug nanocarriers for cancer imaging and therapy.

### Future Perspectives

From the discussion, the future research of the g-C3N4 nano-based compound may focus on synthesizing innovative g-C3N4 nano-based particle which are responsive to morphology monitoring, evaluating the photocatalysis practicality and efficacy of traditional synthesis and preparative strategies of g-C3N4 nano-based compound, and then exploring the applications of diverse g-C3N4 nano-based particles in treating commercial wastewater, its effective application in solar energy utilization, environmental treatment, biomedical and sensing applications by fully assessing their photocatalytic ability, cost, energy consumption, and reusability.

## Conclusions

In conclusion, this mini review climaxes the current advances on the structure and preparation techniques of full-bodied g-C_3_N_4_ nano-based material. Understandably, g-C_3_N_4_ has demonstrated to be one of the greatest favorable entrants suitable for scheming and assembling innovative composite photocatalysts. Thus, there is little uncertainty that the massive advancement of g-C_3_N_4_ nano-based particle will endure to develop in the near future. In view of that, more studies are also needed to making full use of the exceptional structural, synthesis, properties, and the preparation techniques of g-C_3_N_4_ nano-based particle.

## Abbreviations

g-C_3_N_4_: Graphite carbon nitride
TiO_2_: Titanium oxide
ZnO: Zinc oxide
